# Sterol imbalances and cholesterol‐24‐hydroxylase dysregulation is linked to the underlying progression of multiple sclerosis

**DOI:** 10.1111/bpa.70001

**Published:** 2025-03-05

**Authors:** Lauren Griffiths, Kristen Hawkins, Eylan Yutuc, Roberto Angelini, Racheal Fosuah, Manuela Pacciarini, Alison Dickson, Neil Robertson, Laura Childs, Samantha Loveless, Emma Tallantyre, William J. Griffiths, Yuqin Wang, Owain W. Howell

**Affiliations:** ^1^ Institute of Life Sciences 1, Faculty of Medicine, Health and Life Sciences Swansea University Swansea UK; ^2^ Division of Psychological Medicine and Clinical Neurosciences, School of Medicine Cardiff University Cardiff UK; ^3^ Helen Durham Unit University Hospital of Wales Cardiff UK

**Keywords:** cholesterol, cholesterol‐24‐hydroxylase (CYP46A1), mass spectrometry, multiple sclerosis (MS), neurodegeneration, progression, sterols

## Abstract

Disability worsening in multiple sclerosis (MS) is linked to neurodegeneration. Cholesterol homeostasis is essential for normal brain function. CYP46A1, crucial for brain cholesterol turnover and reduced in some neurodegenerative diseases, is a potential neuroprotective target. We hypothesized that CYP46A1 is downregulated in MS brains and linked to cholesterol dysbalance. Mass spectrometric analysis of sterols was performed from matched plasma and cerebrospinal fluid (CSF) in an all‐female MS cohort (*n* = 32, mean age = 33). Disability status was recorded at baseline and follow‐up. MS brain tissue samples (*n* = 11; 7 females; ages 38–67; 10 Secondary Progressive MS, 1 Primary Progressive MS; Disease Duration: 13–49 years) and control samples (*n* = 8; 3 females; ages 41–68) analysed for pathological regions using mass spectrometry and RNA expression using in‐situ hybridization. Significant dysregulation in 25‐hydroxycholesterol, 27‐hydroxycholesterol and 3β‐hydroxycholestenoic acid in CSF correlated with disability at baseline and follow‐up in the patient population. In brain tissue, reduced cholesterol, 24S‐hydroxycholesterol and 24S,25‐epoxycholesterol were observed in white matter lesions (*p* < 0.05), linked to CYP46A1 activity. CYP46A1 expression was enriched in neurons, with reductions in MS grey matter lesions and non‐lesions compared to controls (*p* < 0.01). Cholesterol metabolism is dysregulated in MS and is associated with reduced neuron‐specific CYP46A1 expression. Modulating CYP46A1, a druggable target, may benefit progressive MS.

## Graphical abstract

We have sought to determine the role that cholesterol metabolism plays in disability worsening and its correlation with progressive MS by analysing patient CSF and post‐mortem tissues using cutting edge mass spectrometry approaches. Key sterol metabolites measured in the CSF link with important disability milestones at the very early stages of MS diagnosis. Cholesterol and its main brain sterol, 24S‐hydroxycholesterol (24S‐HC), are dysregulated within the brain tissue itself, and the expression of enzyme involved in the metabolism of cholesterol for excretion, CYP46A1 is downregulated. These novel findings could have implications for MS as CYP46A1 activity can be modulated therapeutically.

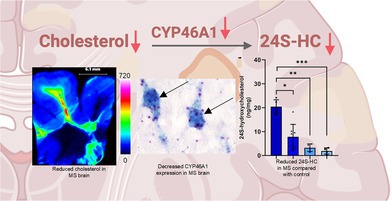

## 1 INTRODUCTION

The progressive accumulation of disability eventually experienced by most people with multiple sclerosis (MS) is underpinned by a combination of inflammatory lesions and neurodegeneration. Monitoring as well as modifying MS progression is hampered by an incomplete understanding of underlying biological mechanisms [1]. Cholesterol, its metabolites, and the enzyme CYP46A1, responsible for cholesterol turnover in the brain, are considered important fluid and imaging biomarkers, as well as potential treatment targets, for a wide range of neurological conditions including MS [2, 3, 4, 5, 6, 7, 8]. Statins have pleiotropic effects and have been shown to modulate inflammatory, oligodendrocyte and neuronal processes [9, 10, 11]. Simvastatin, a brain‐penetrant statin, has been shown to reduce the rate of whole brain atrophy and aspects of neuropsychiatric dysfunction in secondary progressive MS when given at a high dose [12, 13], and supports the growing evidence that the balance between cholesterol synthesis and metabolism is important to biological processes underpinning the progressive accumulation of disability [14]. However, recently, high‐dose simvastatin was found to be ineffective in modifying confirmed disability progression in a larger cohort [15].

Alterations in sterols, a major category of lipids with the fused four‐ring core structure, and the enzymes involved in the cholesterol metabolism pathways, are implicated in MS and other neuroinflammatory and neurodegenerative diseases [16]. Cholesterol's inability to cross the blood brain barrier (BBB) means that it is synthesised and metabolised locally within the central nervous system (CNS), independently of dietary cholesterol, with differences between males and females [17]. Cholesterol homeostasis is essential for normal neuronal and synaptic function, is a vital component of myelin and is a rate‐limiting component for CNS repair mechanisms including remyelination [18]. CYP46A1 (also known as Cholesterol 24‐hydroxylase) is crucial for cholesterol homeostasis as it transforms cholesterol to 24S‐hydroxycholesterol (24S‐HC; cerebrosterol) and allows its removal from the CNS [14]. CYP46A1 also converts desmosterol to 24S,25‐epoxycholesterol (24S,25‐EC) which, alongside 24S‐HC, is a potent ligand to Liver X receptors (LXRs) that are transcriptional activators of genes involved in lipid metabolism and inflammation [19]. Notably, CYP46A1 expression is highly enriched within CNS neurons and choroid plexus epithelia [20], so that systemic levels of 24S‐HC also reflect central cholesterol metabolism, making CYP46A1 and its metabolites, attractive candidate biomarkers of neurological dysfunction [21]. A selective CYP46A1‐targeted positron emission tomography (PET) tracer has shown some promise as a means to monitor cholesterol metabolism and neuron function in vivo [22]. Activation of CYP46A1 is protective in experimental models of Huntington's and Alzheimer's disease, where the rate of cognitive decline can be attenuated [6, 23], and its product, 24S‐HC, alongside other cholesterol metabolites, differs between MS and controls, and between relapsing and progressive MS cohorts [8, 24, 25].

To better understand the role of cholesterol homeostasis and CYP46A1 in relation to MS progression, we have: (i) analysed cholesterol and a selection of sterol intermediates involved in its synthesis and metabolism in both the plasma and cerebrospinal fluid (CSF) from people with MS who later experienced disability; (ii) used mass spectrometry imaging and lipid extraction techniques to show the extent of cholesterol and sterol dysbalance in post‐mortem cases of active progressive MS, and (iii) quantified *CYP46A1* messenger RNA (mRNA) expression in normal and lesioned MS grey matter (GM). Our work reveals altered cholesterol homeostasis in early MS and a reduction in the levels of key sterol intermediates, including 24S‐HC and its biosynthetic enzyme, CYP46A1, in cases of active progressive MS. This study highlights the potential value of monitoring and targeting sterol metabolism to restore normal cholesterol homeostasis in the MS brain.

## 2 MATERIALS AND METHODS

### 2.1 Patient cohort for plasma and CSF analysis

Sex is a key variable in the abundance of cholesterol and related sterols. We removed this variable to improve study power by selecting an all‐female relapsing MS cohort (*n* = 32, mean age of onset = 33; range 16–50), mean age at lumbar puncture (LP) = 37.5 years (range 20–60) of patient‐matched plasma and CSF samples (see Table 1; Research Ethics Committee approval; 19/WA/0289; 24/WA/0049). Plasma and CSF were captured at baseline before the initiation of disease‐modifying treatment. Researchers performing the laboratory measurements were blinded until analysis was complete. Expanded disability status scale (EDSS) at baseline and at follow‐up (average between baseline and most recent EDSS: 90 months (range: 0–193)) was collected as part of standard care. Capture of plasma and CSF followed standard protocol.

**TABLE 1:** Cases with matched plasma and CSF used for sterol analysis (Welsh National Research Tissue Bank).

Case ID	Sex	Age at LP (years)	EDSS (baseline)	Time from baseline to latest EDSS (months)	Latest EDSS	EDSS change	Relapse number at 5 years	First line DMT (or escalated within 5 years)	Diagnosis
7204	Female	35	1	144	4	3	7	Alemtuzamab	RRMS
10821	Female	22	0	193	6	6	3	Plegridy	RRMS
11333	Female	60	<4.0	0	4	0	4	No DMT	SPMS
12025	Female	31	6	10	6	0	3	Aubagio	RRMS
12391	Female	45	4	163	0	–	U/K	No DMT	RRMS
12915	Female	42	3.5	181	4	0.5	6	Alemtuzamab	RRMS
13311	Female	33	<4.0	144	4	0	3	Copaxone	RRMS
13870	Female	44	0	152	2	2	1	No DMT	RRMS
13947	Female	37	<4.0	156	4	0	1	Tecfidera	RRMS
15150	Female	42	4	117	U/K	U/K	1	No DMT	CIS
21510	Female	35	2	125	4	2	4	Tecfidera, esc. to Ocrevus within 5 years	RRMS
25969	Female	50	1	103	6.5	5.5	1	No DMT	RRMS
27835	Female	23	1	25	4	3	4	Avonex, esc. to fingolimod under 3 years	RRMS
28012	Female	28	3	109	4	1	1	Alemtuzamab	RRMS
30277	Female	31	1.5	104	5.5	4	1	Nataluzimab	RRMS
38076	Female	45	0	98	6	6	5	Tecfidera, esc. to fingolimod under 3 years	RRMS
40687	Female	26	2	67	3.5	1.5	3	Nataluzimab	RRMS
41964	Female	51	4.5	95	6.5	2	1	Tecfidera, esc. to Ocrevus under 3 years	RRMS
42345	Female	26	U/K	76	4	4	3	Fingolimod	RRMS
44151	Female	28	2	94	3	1	6	Avonex, esc. to Ocrevus within 4 years	RRMS
44914	Female	38	1	93	3	2	3	Tecfidera, esc. to mavenclad within 5 years	RRMS
46249	Female	43	2	85	6	4	1	Tecfidera	RRMS
68722	Female	32	1	87	4	3	1	Tecfidera	RRMS
70647	Female	46	4	65	6.5	2.5	4	Ocrevus	RRMS
71860	Female	37	<4.0	25	1.5	0	1	Tecfidera	RRMS
71873	Female	41	1	0	1.5	0.5	1	No DMT	CIS
75658	Female	40	1	70	4	3	3	Tecfidera	RRMS
76827	Female	20	3.5	73	4	0.5	4	Alemtuzamab	RRMS
77509	Female	48	6	61	6	0	2	Tecfidera, esc. to Ocrevus within 5 years	RRMS
83565	Female	40	<4.0	67	4	0	5	Ocrevus	RRMS
83643	Female	36	0	69	4	4	4	Ocrevus	RRMS
84390	Female	45	1	60	1	0	2	Tecfidera, esc. to cladribine under 1 year	RRMS

Abbreviations: −, number below zero; CIS, clinically isolated syndrome; DMT, disease‐modifying therapy; EDSS, expanded disability status scale; esc., escalated; LP, lumbar puncture; RRMS, relapsing–remitting MS; SPMS, secondary progressive MS; U/K, unknown.

### 2.2 Post‐mortem tissue

Snap‐frozen human brain tissue blocks were provided by the UK MS Society Tissue Bank (MSSTB) (Imperial College, London, UK) for MS tissue, and the Thomas Willis Brain Bank (Oxford University, Oxford, UK) for the non‐neurological control tissue, with appropriate ethical approval (research ethics committee approvals 08/MRE09/31 + 5 and 13/WA/0292; see Table 2 for full cohort demographics). All MS cases were confirmed as either secondary (SPMS) or primary (PPMS) progressive MS at the time of death (MS: *n* = 11; 7F; 1 PPMS, age at death: 38–67); (control: *n* = 8; 3F; age at death: 41–68). Sample availability and the presence of pathological and non‐lesion areas of interest determined the cases used in this arm of the study.

**TABLE 2:** MS and control post‐mortem cases for in situ hybridisation, region enriched homogenates (marked #) and MALDI cholesterol analysis (marked *).

Case number	Sex	Age	PMD (h)	Cause of death	MS subtype	Disease duration (years)
MS402 #	Male	46	12	Bronchopneumonia, MS	SPMS	20
MS407 #	Female	44	22	Septicaemia, bronchopneumonia	SPMS	19
MS423 #*	Female	54	10	Bronchopneumonia	SPMS	30
MS438 #	Female	53	17	MS	SPMS	18
MS461 #	Male	43	13	Bronchopneumonia	SPMS	21
MS473 #*	Female	39	9	Bronchopneumonia, MS	PPMS	13
MS510 #*	Female	38	19	Bronchopneumonia, MS	SPMS	22
MS513 #*	Male	51	17	Respiratory failure, MS	SPMS	18
MS530	Male	42	15	Bronchopneumonia, MS	SPMS	21
MS538	Female	62	12	Pancreatic cancer	SPMS	31
MS541 #	Female	67	U/K	U/K	SPMS	49
NP13/011 #*	Female	62	24	Metastatic colorectal cancer	n/a	n/a
NP13/012 #*	Female	60	48	Metastatic breast cancer	n/a	n/a
NP13/039	Male	41	24	Myocardial infarct	n/a	n/a
NP13/073	Male	59	24	Chronic obstructive pulmonary disease	n/a	n/a
NP13/103	Female	48	56	End‐stage interstitial lung disease	n/a	n/a
NP13/126 #	Male	56	40	Cardiac arrest	n/a	n/a
NP13/127 #	Male	60	30	Cardiac arrest	n/a	n/a
NP13/128 #*	Male	68	48	Cardiac arrest	n/a	n/a

Abbreviations: DD, disease duration; n/a, not applicable; PMD, post‐mortem delay; PPMS, primary progressive MS; SPMS, secondary progressive MS; U/K, unknown.

### 2.3 Determining plasma GFAP and NfL concentrations

Highly sensitive single molecule array (SiMoA) technology was employed to quantify neurofilament light (NfL) and glial fibrillary acidic protein (GFAP) in patient plasma samples (*n* = 32). Samples were quantified on the Quanterix HD‐X analyser using single‐plex bead‐based assays: Human NF‐light v2 Advantage (cat#104073) and GFAP Advantage Plus (cat#104619), following the manufacturers protocol.

### 2.4 Histology, immunohistochemistry and tissue characterisation

Sequential 6 mm thick cryosections were thawed, fixed in 4% paraformaldehyde and processed for histology (Luxol fast blue; LFB) and/ or immunostaining (and detected using the IMPRESS‐peroxidase detection kit with diaminobenzidine as the chromogen, Vector Labs). Sequential cut slides were stained for LFB/ mouse anti‐Human HLA‐DP, DQ, DR Antigen (HLA; clone: CR3/ 43, Agilent Technologies Inc.) and mouse anti‐myelin oligodendrocyte glycoprotein (MOG, clone: Y10, R. Reynolds, Imperial College London) to detail tissue anatomy and to classify demyelinated lesions as: active (characterised by a demyelinated area confluent with HLA+ microglia/ macrophages); chronic active (termed ‘smouldering’, ‘slowly expanding’ or ‘mixed active/ inactive’ by others, characterised by a demyelinated lesion centre and a border of HLA+ microglia/ macrophages containing LFB+ phagocytosed membranes); chronic inactive [hypocellular lesion centre and a rim of HLA+ cells indistinguishable in number from the surrounding normal appearing (NA) white matter (WM)], or remyelinated (completely remyelinated shadow plaque) [26]. Cortical GM lesions (GML) were described as leukocortical, intracortical or subpial [27]. Immunohistochemistry for CYP46A1 (mouse Anti‐Cholesterol‐24 Hydroxylase, clone: 1A7; Merck) was used to identify protein expression of the enzyme in representative cases of MS and control donor brain tissue. Single immunostained sections were counterstained with Gill's haematoxylin (Vector). All sections from all cases were stained as part of the same experimental run and appropriate negative and positive staining controls were included. Images were captured using a Zeiss Axio Scope 1 at 100–630x magnification fitted with a Zeiss MRm 503 colour camera or with the Zeiss Axio Scanner 1.

### 2.5 In situ hybridisation for CYP46A1

*CYP46A1* (NM_006668.1; RNAscope Probe—Hs‐CYP46A1m) RNA expression was detected by in situ hybridisation (RNAscope ISH from Advanced Cell Diagnostics, Bio‐Techne, Minnesota, USA) on snap‐frozen MS and control tissue sections, with the full methodology described in Supporting Information. Specific probe binding was revealed by chromogenic (FastRed) development. All sections were counterstained in haematoxylin. For analysis, four x400 magnification images were taken per region of interest (control GM or MS normal and lesion GM) per case, and the average percent positive signal captured in QuPath [28, 29] and expressed relative to the number of transcript‐positive cells to account for differences in cell density between regions of interest.

To explore *CYP46A1* expression in neurons and astrocytes, we combined in situ hybridisation for *CYP46A1* with immunohistochemistry for neurons (anti‐HUC/D, Invitrogen) and astrocytes (anti‐GFAP, DakoCytomation). Additionally, we used CellxGene VIP (https://cellxgenevip-ms.bxgenomics.com/), which is based on a single nucleus RNAseq study of control and MS donor cortex [30], to visualise *CYP46A1*, *RBFOX3* and *ELAVL3* gene expression by cell type.

### 2.6 Sterol analysis

Sterol analysis from biofluid and tissue homogenate supernatant was performed as stated [31] using enzyme‐assisted derivatisation for sterol analysis (EADSA) with an additional hydrolysis step performed on all CSF samples analysed. Matrix‐assisted laser desorption‐ionisation (MALDI) imaging of cholesterol was performed as described [32], with minor modifications (see Table 2 and Supporting Information). For tissue homogenisation, pathological regions of interest—chronic active WM lesions, cortical GM lesions, and NAWM and GM (blocks chosen based on WM pathology, with GM pathology dissected only if GM lesions were present), where available, were manually dissected from 15 mm cryosections per block until ~2 mg of each region of interest was collected (*n* = 9 for MS, *n* = 5 for control tissue) and homogenised using Precellys single‐use ceramic bead homogenate tubes (PN: P000912‐LYSK0‐A, Bertin Technologies, France) with a sterol internal standard‐ethanol mix (Supporting Information Table S2) for relative quantification. The supernatant‐standard mix underwent the EADSA protocol before analysis by high‐performance liquid chromatography (HPLC) mass spectrometry with multi‐stage fragmentation using an Ultimate 3000 HPLC system (Dionex) coupled with an Orbitrap Elite hybrid high‐resolution mass spectrometer (ThermoFisher Scientific).

### 2.7 Statistical analysis

All statistical analysis was performed using GraphPad Prism 10 software. The majority of the data were non‐normally distributed, and non‐parametric analyses were used throughout. Spearman correlation analysis was used to explore the inter‐relationships between sterol levels and clinical measures. Multiple‐linear regression, accounting for age as the co‐variant, was used to investigate the relationship between sterol levels and EDSS at follow‐up for those sterols that associated with age at LP (patient cohort). Kruskal–Wallis with false discovery rate correction was used for comparing three or more groups. Data were plotted as scatter graphs with bars representing mean values (± standard deviation) per region of interest per case.

## 3 RESULTS

### 3.1 The levels of key sterol metabolites in the CSF are associated with later disability

**FIGURE 1:**
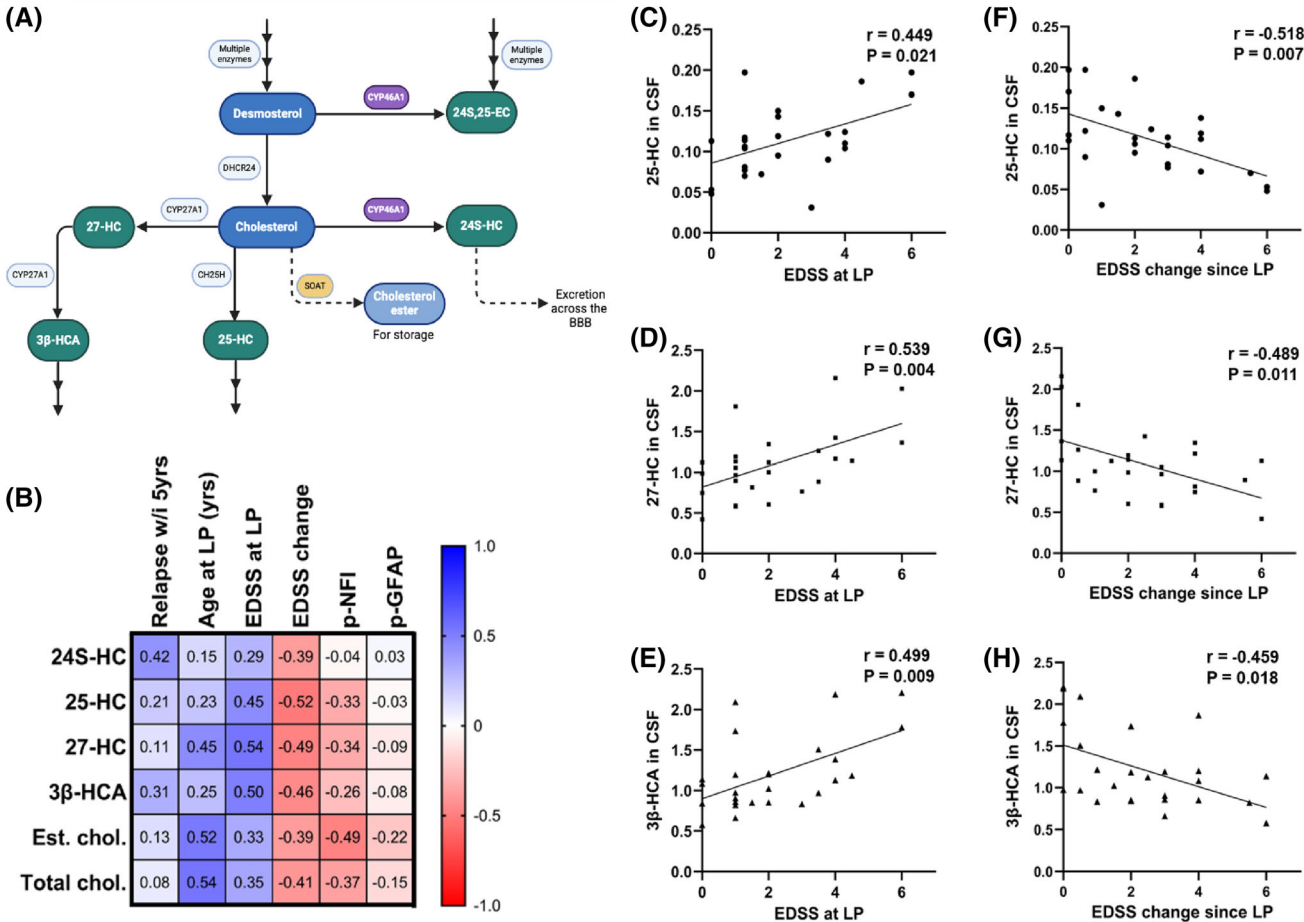
The association between sterol metabolites in the CSF and clinical measures of progression. (A) Bloch pathway schematic depicting cholesterol synthesis and metabolism in the brain. (B) Spearman correlative analysis revealed associations between 25‐HC, 27‐HC (also known by the more systematic name (25R)26‐hydroxycholesterol) and 3β‐HCA with EDSS at the time of lumbar puncture (LP) (C, D and E, respectively). Levels of 25‐HC, 27‐HC and 3β‐HCA correlated with EDSS change at follow‐up (F, G and H, respectively). Spearman *r* values are shown in a matrix with separate correlation graphs showing Spearman *r* and exact *p* values. EC, epoxy‐cholesterol; HC, hydroxycholesterol; HCA, hydroxycholestenoic acid; w/i, within; yrs, years; p‐NFl, plasma neurofilament; p‐GFAP, plasma glial fibrillary acidic protein; est., esterified; chol., cholesterol; CSF, cerebrospinal fluid.

We first investigated sterol homeostasis in patient CSF and plasma by mass spectrometry analysis (pathway shown in Figure 1A). Spearman correlation analysis was used to explore associations between sterol levels and clinical milestones. Neither sterol, NfL nor GFAP levels in MS plasma associated with any of the clinical information provided. In CSF, levels of 25‐HC, 27‐HC and 3β‐HCA (ng/ml) correlated with EDSS at baseline (*r* = 0.449 to 0.539; *p* = 0.021 to 0.004; Figure 1B–E). CSF 25‐HC, 27‐HC and 3β‐HCA levels modestly associated with the change in EDSS at follow‐up (*r* = −0.459 to −0.518, *p* = 0.007 to 0.018; Figure 1B,F–H), which survived multiple‐linear regression testing when adjusting for age at LP (*p* = 0.0042, 0.0043 and 0.012, respectively). Additionally, the CSF concentration of total and esterified (stored) cholesterol correlated with age at LP (*r* = 0.537 and 0.519; *p* = 0.002 and 0.002, respectively). Levels of esterified cholesterol correlated with plasma NfL (Figure 1B; *r* = −0.488, *p* = 0.005). This exploratory correlation analysis on a small cohort of patient samples at a single timepoint revealed altered sterol homeostasis in early MS, and its association with later disability worsening.

### 3.2 Cholesterol and 24S‐HC are significantly reduced in the MS brain

To better understand the association between cholesterol metabolism and pathology, we next quantified sterol levels in brain tissue from autopsy cases that experienced an active progressive course.

**FIGURE 2:**
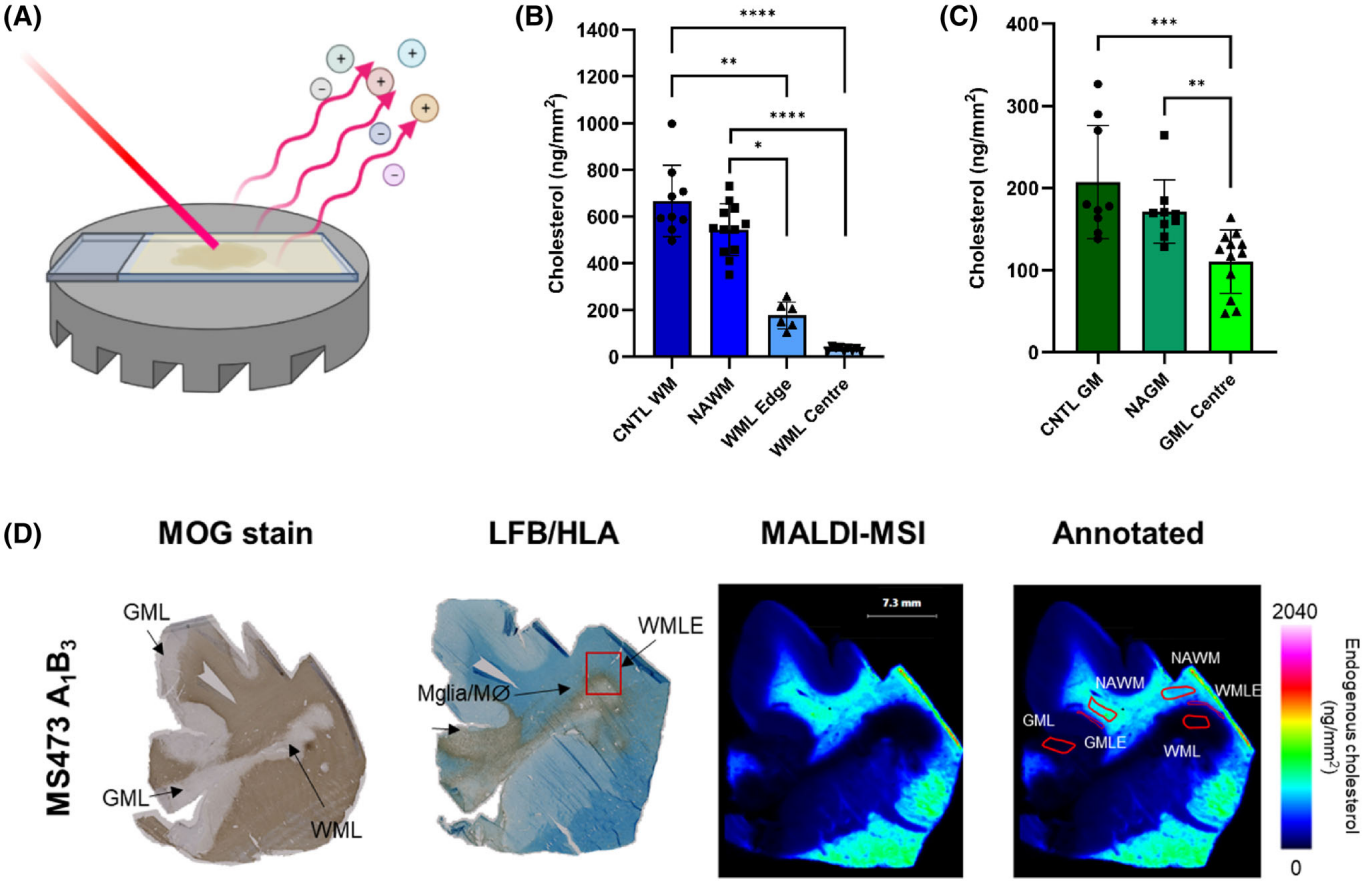
Mass spectrometry imaging (MSI) technique shows significant depletion of cholesterol in MS tissue. The spatial visualisation and quantification of cholesterol using MSI technology coupled with matrix‐assisted laser desorption/ionisation (MALDI) of MS and control human brain tissue (A), with significantly decreased levels of cholesterol in regions of interest in the white matter (B), and grey matter (C; mean with SD). The visualisation of the spatial distribution in the normalised images (D) show subtle cholesterol differences that cannot be picked up with standard immunohistochemical techniques.

**FIGURE 3:**
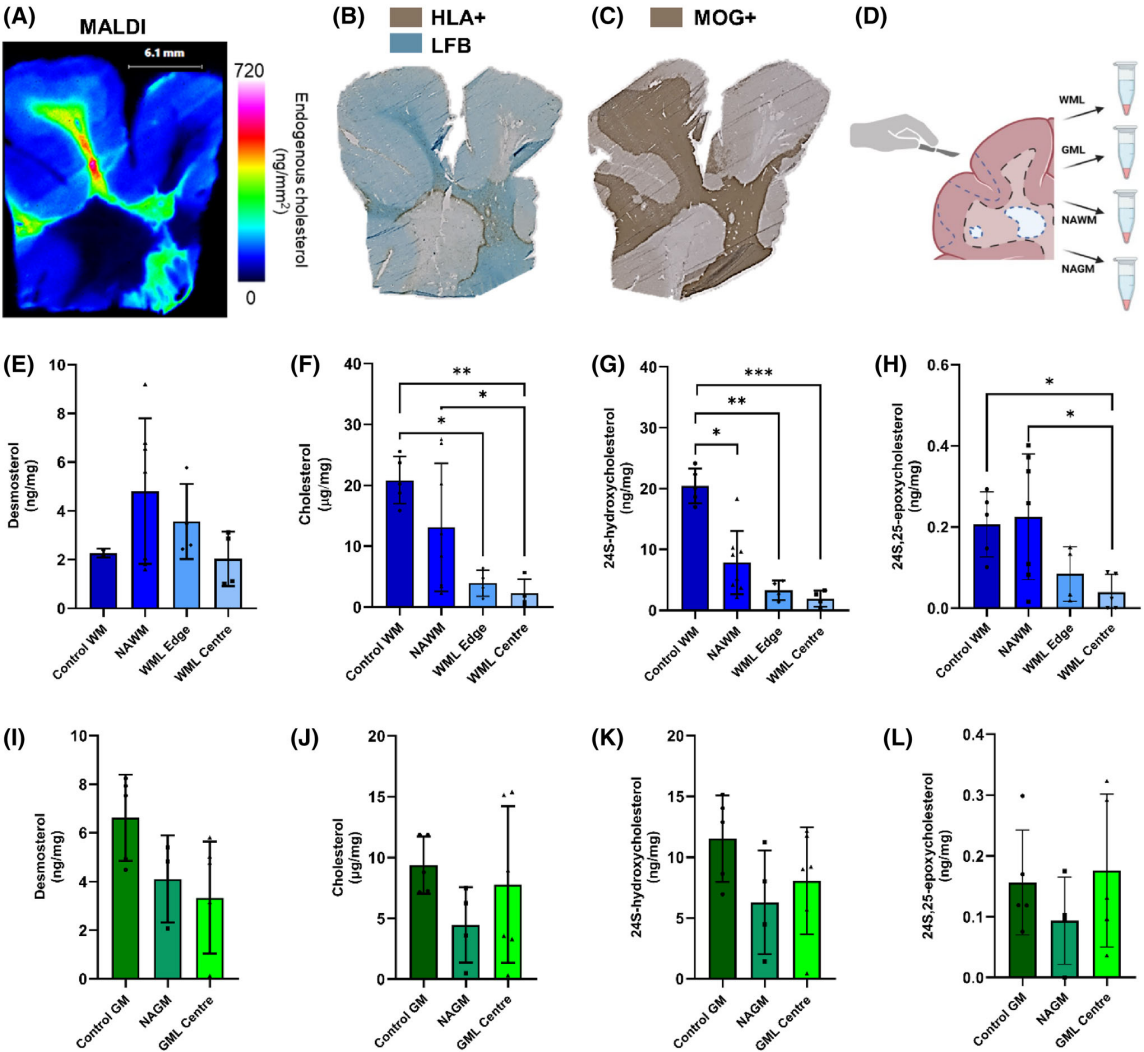
Significant dysregulation of key sterol metabolites in the white matter of the MS brain. Analysis of cholesterol using matrix‐assisted laser desorption/ionisation (MALDI) imaging (A) showed large difference in pathological regions of interest and supported the need for further sterol analysis of brain tissue. Dual staining of LFB /HLA (B) and MOG‐stained tissue (C) guided the dissection for region‐enriched homogenisation (D). Data from the white matter (E–H) showed significant changes in sterol metabolites, measured by liquid chromatography‐mass spectrometry for relative quantification (Kruskal–Wallis and false discovery rate correction). There was a trend to a difference in the concentration of the same sterols in MS grey matter (I–L; *n* = 3–5 MS regions of interest, *n* = 6 for control). WM, white matter; NAWM, normal appearing white matter; WML, white matter lesion; GM, grey matter; NAGM, normal appearing grey matter; GML, grey matter lesion.

A sub‐selection of cases was initially analysed by a MALDI‐mass spectrometry imaging (MSI) technique [32] optimised to visualise cholesterol in human brain tissue (Figure 2), with cholesterol measured in different regions of interest, as shown in the annotated image in Figure 2D. The MALDI‐MSI analysis revealed significantly altered cholesterol levels in both the GM and WM pathological regions of the MS brain (Figures 2B–D and 3A).

To further investigate cholesterol and the less abundant sterols, such as 24S‐HC, which cannot be uniquely identified by MALDI‐MSI, we analysed region‐enriched homogenates representing NAWM, chronic active WM lesion (WML) edge, WML centre (taken from the centre of either an inactive or chronic active lesion), NAGM and GML centre (where available), and anatomically matched control WM and GM (Figure 3B–D). The resulting sterol analysis by EADSA LC–MS showed significant differences in sterol levels of MS WML (Figure 3E–H) in comparison to control and NAWM.

Cholesterol was significantly reduced throughout the WM in MS brain (Figure 3F), showing a decrease in WML centre and WML edge compared with control (*p* = 0.0035 and *p* = 0.0279, respectively). Additionally, cholesterol in WML centre was decreased compared to NAWM (*p* = 0.0232). 24S‐HC was decreased in WML centre (*p* = 0.0005), WML edge (*p* = 0.0046) and NAWM (*p* = 0.039) in comparison to control WM. 24S,25‐EC was increased in NAWM compared with control (Figure 3H). The levels of 24S,25‐EC were reduced in WML centre compared with both control WM (*p* = 0.0144) and MS NAWM (*p* = 0.0147). NAGM and GML samples were prepared from the same blocks as selected for the WM analysis, but the number of coincident GMLs was low and only six of the sampled blocks contained sufficient areas (NAGM or GML centre) for homogenisation. There was a similar trend of lower sterol concentrations in the GM regions in MS in comparison to control and between GM lesion and NAGM (Figure 3I–L); however, no significance was observed.

### 3.3 Expression of CYP46A1, a neuronal enzyme key for cholesterol regulation and transport, is reduced in MS brain

**FIGURE 4:**
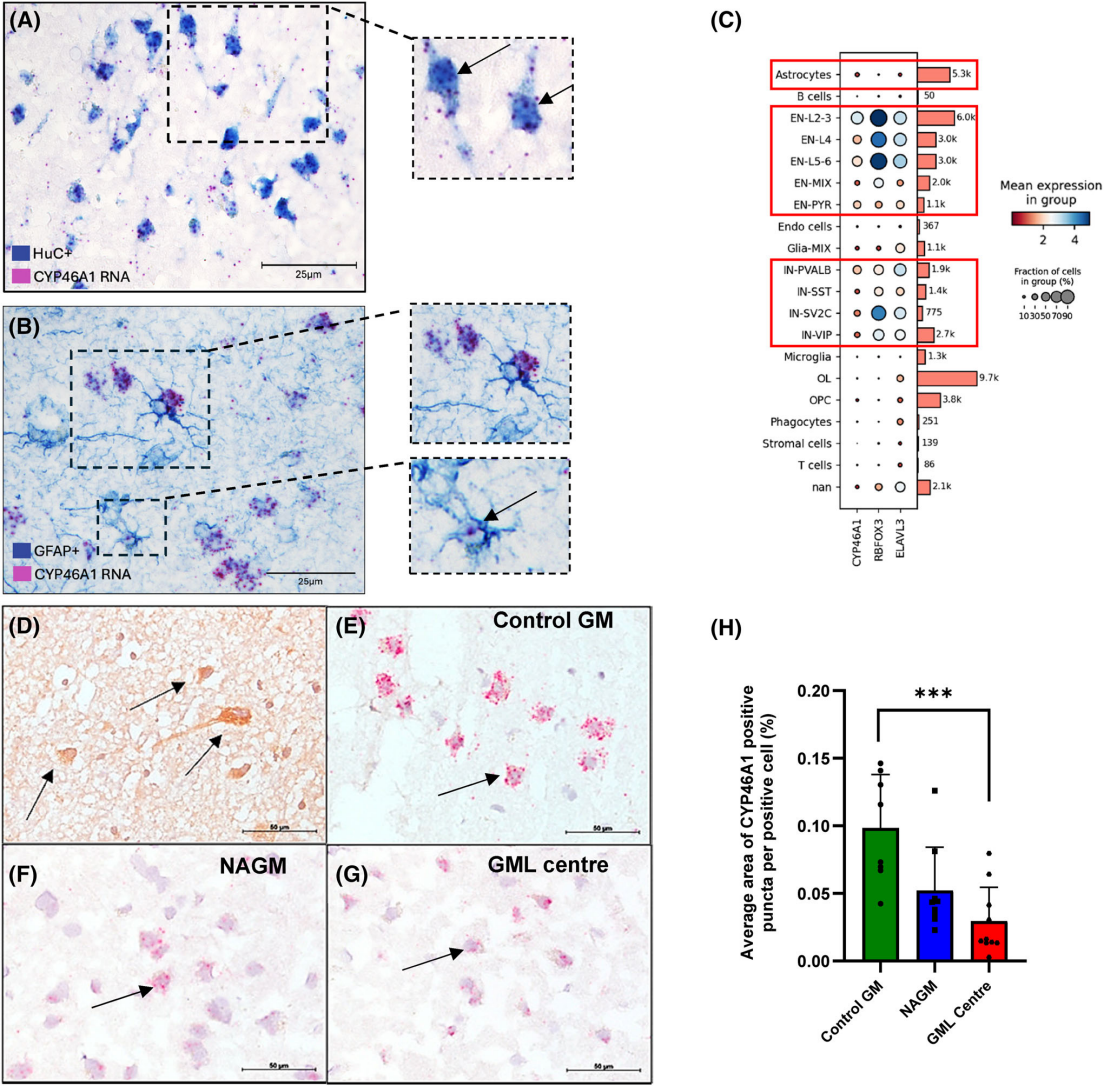
*CYP46A1* expression is significantly reduced in the MS brain. (A, B) Dual in situ hybridisation/immuno‐staining revealed *CYP46A1* expression by HuC+ neurons, with limited expression in GFAP+ astrocytes (arrow, B). (C) Single nucleus transcriptomic data (Schirmer et al. [30]) showing *CYP46A1* expression enriched in the same cell types as RBFOX3 (NeuN) and ELAVL3 (HuC). CYP46A1 in inhibitory (lower boxed area) and excitatory neurons (middle box), with very little in astrocytes (upper box). (D) CYP46A1 antibody staining (arrows, brown reaction product) confirmed protein expression in the NAGM tissue. In situ hybridisation revealed *CYP46A1*+ puncta decorating large neuron‐like cells of the control and MS cortical GM (arrows; E–G), and decreased *CYP46A1* transcript expression in GML (H; comparing the average area of transcript positive puncta per positive cell; Kruskal–Wallis and false discovery rate correction). GM, grey matter; NAGM, normal appearing grey matter; GML, grey matter lesion. Scale bars: A, B = 25 μm, D–G = 50 μm.

As the depletion of cholesterol, 24S‐HC and 24S,25‐EC in MS brain white matter suggested a potential metabolic impairment, we investigated the expression of *CYP46A1* in donor brain tissue by in situ hybridisation and immunostaining. A majority of *CYP46A1* puncta revealed by in situ hybridisation associated with the neuronal antigen HUC/D+ (neuronal marker) but not GFAP+ (astrocyte) cells (Figure 4A,B), and was further supported by an mRNA transcriptomic dataset, showing *CYP46A1* expression in a similar subset of cells as seen for established markers of mature CNS neurons: NeuN (RBFOX3) and HUC (ELAVL3; Figure 4C). Anti‐CYP46A1 immunohistochemistry confirmed a neuron‐like localisation of CYP46A1 protein (Figure 4D). There was a significant reduction of *CYP46A1* mRNA expression per cell (percent area of positive puncta relative to the total number of positive cells) in GML areas (0.029% ± 0.025; *p* = 0.0009) compared with anatomically matched control GM (0.098% ± 0.040) (Figure 4E–H). In addition, there was a trend to a reduced *CYP46A1* expression in MS NAGM (0.052% ± 0.032) compared with control GM (*p* = 0.064), and between MS GML and NAGM (*p* = 0.083).

## DISCUSSION

We show that cholesterol homeostasis is dysbalanced early in MS and is associated with later disability. Cholesterol and its metabolites are reduced at sites of inflammatory demyelination, and the expression of CYP46A1, a key enzyme in maintaining cholesterol homeostasis, was reduced in the MS GM. Given that restoring cholesterol homeostasis is associated with neuroprotection and that new tools for the modulation and in vivo imaging of CYP46A1 activity are being developed, our data suggest that CYP46A1 could represent a treatment target or its activity a biomarker of underlying pathological processes that contribute to MS progression.

While prior studies have investigated sterol levels in blood and CSF, comparisons have been made to control and between different clinical subgroups [8, 24, 33, 34] but not with measures of tissue pathology that partly drive progression. Our exploratory correlation analysis of sterols in matched patient plasma and CSF from the time of diagnosis showed significant associations between CSF sterols and current and later disability. Note that an all‐female group was used for the patient analysis to account for known sex differences in sterols. A future analysis of male‐donor fluid samples would be needed, particularly given the small differences in progression and underlying pathology seen with sex [35, 36]. 25‐HC plays an important role in inflammation, with the enzyme responsible for its synthesis (CH25H) found to be expressed in activated macrophages, and the metabolite itself acting as an amplifier of inflammation [14, 37]. 27‐HC is an abundant circulating oxysterol generated from the metabolism of free cholesterol and a precursor to the synthesis of the bile acids, like 3β‐HCA [14]. 27‐HC is a ligand for oestrogen receptors, important in remyelination, while bile acids can affect neuroinflammatory processes and are reduced in MS plasma [38]. Bile acid supplementation can ameliorate experimental autoimmune encephalomyelitis [38]. 3β‐HCA has been linked with motor neuron loss in mouse models, suggesting neurotoxic effects [39]. Whether the altered sterol profile noted here is a reliable prognostic biomarker for progressive disability warrants further investigation in larger patient groups.

Disrupted cholesterol homeostasis is associated with neurodegenerative and age‐related neuropathology [40], while age and ageing play a determining role in the MS course [41]. Our finding that CSF cholesterol correlates with age agrees with previously published data [42]. A further investigation of the balance between central cholesterol synthesis and metabolism in younger and older people with MS would be of interest given the known age‐associated changes in both cholesterol metabolism and MS. Therefore, restoring cholesterol homeostasis in the ageing MS brain might be a therapeutic goal as it is for neurodegenerative diseases such as AD and PD [43].

We were able to show that cholesterol metabolites 24S‐HC and 24S,25‐EC are depleted in the WM of MS brain donors characterised by an active pathology. These findings were confirmed by the application of two different mass spectrometry methodologies, both MALDI‐MSI on‐tissue (for cholesterol) and HPLC‐MS analysis from the regional homogenates. MALDI mass spectrometry imaging demonstrated subtle alterations in cholesterol, which are not readily apparent in standard histology [44]. Changing myelin lipid composition can affect axo‐glial interactions, where alterations in the architecture of the node of Ranvier are a frequent feature of the MS NAWM and are predicted to hamper normal neural transmission [45]. Oxysterol‐LXR interactions serve to modulate inflammatory, lipid metabolic and astroglial responses [46], meaning that disrupted cholesterol homeostasis and reduced levels of 24S‐HC and 24S,25‐EC could further neuroinflammatory and neurotoxic effects in the MS brain.

Our findings of reduced *CYP46A1* gene expression in progressive MS show that transcript expression is downregulated in parallel with sterol depletion. In addition to transcriptomic data showing expression is enriched in inhibitory and excitatory neurons, new data show *CYP46A1* is also enriched in the choroid epithelia [20], where its activity is modulated in part by TNF‐receptor1 signalling. TNF signalling is disrupted in progressive MS characterised by focal immune cell aggregates in the leptomeninges, and this disrupted TNF‐receptor1 activation drives a necroptotic form of neuron cell death [47]. Therefore, reduced neuronal *CYP46A1* expression and altered 24S‐HC levels in the CSF could be indicative of neural cell dysfunction because of TNF‐mediated pathophysiological processes.

Restoring CYP46A1 activity and cholesterol homeostasis through allosteric modulation or viral vector delivery is a therapeutic goal in Huntington's disease (ClinicalTrials.gov: NCT05541627). Efavirenz, an FDA‐approved drug, increases CYP46A1 activity at doses below those needed for its anti‐viral effect, is associated with improvements in cholesterol turnover in cell models, animal models and humans [21]. In addition to its capability of modulation via therapeutics, CYP46A1 activity can be monitored by PET [22], using the radiotracer ^18^F‐cholestify, although how radiotracer binding relates to the abundance of 24S‐HC in CSF or serum has yet to be determined. It may be that PET studies could, in the future, reveal those MS patients with reduced CYP46A1 activity who may be more at risk of neurological progression or might be prioritised for neuroprotective treatment targeting the cholesterol pathway.

This work is not without limitations. Given the exacting mass spectrometry approaches used, we were limited in study size and larger clinical and autopsy cohorts might in the future reveal other sterol metabolites or precursors to cholesterol in dysbalance and help us better understand the differences between sterol metabolism in males and females. Our GM homogenate analysis was underpowered to detect differences, such as those seen in the WM. Analysing cholesterol metabolism in MS GM would be important, as cortical lesions represent the predominant lesion type of progressive MS [48].

Our work demonstrates that changes in cholesterol homeostasis, including a reduction in *CYP46A1* expression, are associated with disease activity and severity. We confirm, through pathological association, that CYP46A1 and its metabolites could be a potential biomarker to monitor aspects of the underlying pathophysiology as well as representing a possible treatment target for MS progression.

## AUTHOR CONTRIBUTIONS

The study was conceived and supervised by W.J.G, Y.W, and O.H; all authors contributed to the acquisition and/ or analysis of data; L.G, K.H, S.L and O.H prepared figures and wrote the first draft; all authors reviewed manuscript drafts and the final submitted version. [Correction added on 7 May 2025, after first online publication: Author contributions have been corrected.]

## FUNDING INFORMATION

This work was supported by the UK MS Society [grant 94], the Research Wales Innovation Fund, the BRAIN Unit Infrastructure Award (Grant no. UA05; funded by Welsh Government through Health and Care Research Wales), MRC Impact Acceleration Account, BBSRC grant no. BB/N015932/1, BB/S019588/1, BB/L001942/1, BB/T018542/1, and by the European Union, as part of the Welsh Government‐funded Academic Expertise for Business project.

## CONFLICT OF INTEREST STATEMENT

The author(s) declared the following financial interests/personal relationships which may be considered as potential competing interests: WJG and YW are listed as investors on the patent ‘Kit and method for quantitative detection of steroids’ US9851368B2. WJG, EY and YW are shareholders in CholesteniX Ltd.

## ETHICS STATEMENT

Are detailed in the methods section.

## Supporting information


Data S1.

## Data Availability

The data that support the findings of this study are available from the corresponding author upon reasonable request.
