# Supplementary material for: Sterol imbalances and cholesterol‐24‐hydroxylase dysregulation is linked to the underlying progression of multiple sclerosis
Source: Brain Pathol. 2025 Mar 5;35(5):e70001. doi: 10.1111/bpa.70001 (PMC12352928; doi:10.1111/bpa.70001)
Supplement: Supplementary file 1 — Data S1. [file BPA-35-e70001-s001.docx]

**Sterol imbalances and CYP46A1 dysregulation is linked to the underlying progression of multiple sclerosis**

Lauren Griffiths^1^, Kristen Hawkins^1^, et al.

**Supplementary Tables**

**Supplementary Table 1:** Amplification steps, incubation time and temperatures for RNAscope analysis of CYP46A1.

**Supplementary Table 2:** Deuterated internal standard concentrations used for the quantification of sterols from all human material analysed.

**Supplementary Methods**

In situ hybridisation methodology for *CYP46A1.*

Matrix-assisted laser desorption/ionisation (MALDI) mass spectrometry imaging tissue preparation and analysis.

| **Amp Number** | **Time (mins)** | **Temperature (°C)** |
| --- | --- | --- |
| 1 | 30 | 40 |
| 2 | 15 | 40 |
| 3 | 30 | 40 |
| 4 | 15 | 40 |
| 5 | 30 | RT |
| 6 | 15 | RT |

**Table S1.** **Amplification steps, incubation time and temperatures for RNAscope analysis of *CYP46A1*.** RT – room temperature.

| **Sterol standard (common name)** | **Sample Type** | **Conc. (ng/μL)** | **Volume per sample (μL)** | **Product number** |
| --- | --- | --- | --- | --- |
| [^2^H_7_]22R-hydroxycholest-4-en-3-one | Brain homogenate | 5 | 200 | 700052P |
| [^2^H_7_]22S-hydroxycholest-4-en-3-one | Plasma | 5 | 1 | 700051P |
|  | CSF | 5 | 0.20 |  |
| [^2^H_7_]24R/S-hydroxycholesterol | Brain homogenate | 4 | 200 | 700018P |
| [^2^H_6_]24R/S-hydroxycholesterol | Plasma | 51.95 | 0.192 | LM-4110 |
|  | CSF | 51.95 | 0.038 |  |
| [^2^H_7_]Cholesterol | Plasma | 1000 | 20 | 700041P |
|  | CSF | 1000 | 0.20 |  |
|  | Brain homogenate | 200,000 | 1000 |  |
|  | MALDI | 200 | 1500 |  |
| [^2^H_6_]Desmosterol | Plasma | 100 | 0.60 | 700040P |
|  | CSF | 100 | 0.03 |  |

**Table S2. Deuterated internal standard concentrations used for the quantification of sterols from all human material analysed.** All standards used were supplied by Avanti Polar Lipids (Alabaster, AL, USA).

**Supplementary Methods**

**In situ hybridisation methodology for *CYP46A1***

The RNAscope^™^ ISH technology (Bio-Techne, Minnesota, US) was used for the analysis of CYP46A1 expression in the MS and control human tissue cohort. Tissue sections were thawed and fixed with 4% paraformaldehyde (PFA) for 30 minutes, followed by a water rinse and dehydration through a series of ethanol concentrations (50%, 70%, 100%) and dried for 15 minutes at room temperature (RT) before an additional 15 minutes at 37°C. The endogenous peroxidase activity in the tissue was quenched with a 0.6% hydrogen peroxide solution in distilled water (ddH_2_O) for 8 minutes at RT, followed by ddH_2_O washes. Tissue sections were then incubated with RNAscope^™^ Protease IV solution (Advanced Cell Diagnostics (ACD), Bio-Techne) for 20 minutes at RT followed by PBS washes. Sections were then incubated with the RNAscope^™^ probe Hs-CYP46A1 for 2 hours at 40°C in a HybEZ II oven (ACD, Bio-Techne). Following incubation, the sections were washed with 1X wash buffer (WB) and stored overnight in a 5x concentration saline-sodium-citrate (SSC) buffer.

The subsequent day begins with washes followed by a series of amplification using reagents provided in the RNAscope^™^ 2.5 HD Detection Reagents RED kit (PN: 322360; ACD, Bio-Techne - see Table S4 for amplification details). Between each Amp step the tissue was washed with WB. Following the final amplification and wash step, the sections were incubated with Fast Red (1 in 60 dilution of Fast Red B into Fast Red A) chromogenic substrates to visualise the mRNA puncta (development time ranging between 2 – 12 minutes), where the reaction was stopped with water. The sections were counterstained with Nissl stain Gill’s haematoxylin for 45 seconds, airdried for 5 minutes at RT, a further 25 minutes at 37°C before mounting using VectaMount permanent mounting medium (Vector Laboratories, California, US). Images were captures at x40 magnification using the Zeiss AxioScope microscope with the Zeiss 503 colour camera. Four images were taken from each region of interest and analysed using QuPath. The data reported used average number of positive puncta per cell.

**Matrix-assisted laser desorption/ionisation (MALDI) mass spectrometry imaging tissue preparation and analysis**

The tissue section for analysis (on ITO glass slides) was removed from -80°C storage and immediately placed into a vacuum desiccator with silica gel beads to prevent condensation on the tissue section. After the tissue was free of surface moisture, the sections were placed into a SunCollect pneumatic sprayer system and sprayed with 200 ng/μL [^2^H_7_] cholesterol isotope-labelled standard in absolute EtOH (54 layers; 20 μL/min flowrate; 900 mm/min velocity; 2 mm line distance; final density of 120 ng/mm^2^). Following the isotope-labelled standard, cholesterol oxidase enzyme was sprayed in 5 mM potassium phosphate (KH_2_PO_4_) buffer at pH 7.0 at a final concentration of 0.264 units/mL (18 layers; 1^st^ layer 10 μL/min, 2^nd^ layer 15 μL/min, all other layers 20 μL/min flowrate; 900 mm/min velocity; 2 mm line distance; final density of 52.8 μU/mm^2^). The slides were incubated in a plastic chamber above 30 mL of pre-warmed HPLC- grade water to create humid conditions for the oxidation reaction to occur for one hour at 37°C, then placed into a desiccator under vacuum to remove excess moisture on the slide. Once dry, the slide was again placed into the sprayer system and sprayed with a [^2^H_0_] Girard-P solution in delivery solvent (70% MeOH, 5% glacial acetic acid), at a final concentration of 0.5 mg/mL (18 layers; 1^st^ layer 10 μL/min, 2^nd^ layer 15 μL/min, all other layers 20 μL/min flowrate; 900 mm/min velocity; 2 mm line distance; final density of 0.1 μg/mm^2^). The slides were again incubated within a chamber above 10 mL of pre-warmed incubation solvent (50% MeOH, 5% glacial acetic acid) at 37°C for one hour. After incubation, the slides were removed from the chamber and placed in the desiccator under vacuum to remove excess moisture. The matrix used for analysis was a-cyano-4-hydroxycinnamic acid (CHCA) in a 3:4:3 mix of water/IPA/ACN at a final concentration of 5 mg/mL (16 layers; 80 μL/min flowrate; 1200 mm/min velocity; final density of 2.66 μg/mm^2^). A separate SunCollect pneumatic sprayer system was used to spray on the matrix.

The instrumentation used for all analysis was a combination of the AP-MALDI from MassTech and an Orbitrap ID-X tribrid mass spectrometer (Thermo Fisher, UK). All data used within this paper was acquired at 50 μm spatial resolution, used the same Orbitrap method acquiring the RAW data in full scan (FT) in positive-ion mode at a resolution of 120,000 over *m/z* range 400-1000 with all data quantified within a mass window of 10 ppm, with ion-trap fragmentation data (MS^3^) using a central mass of *m/z* 521.9 with a wide isolation window of 8 mass units. This allowed for the simultaneous fragmentation of both endogenous cholesterol (*m/z* 518.4105 ± 5 ppm) and the isotope-labelled standard (*m/z* 525.4544 ± 5 ppm). All MALDI data was analysed using Multimaging (Imabiotech, France). This software was used for the normalisation of data to the isotope-labelled standard to create a normalised image of cholesterol (*m/z* 518.4105 ± 5 ppm normalised to *m/z* 525.4544 ± 5 ppm) displayed as percentage of normalised intensity, and to provide accurate quantification of cholesterol. It was also used for the quantification of cholesterol in specific regions of interest using the Region of Interest (ROI) tab. The ROI was marked freehand on the tissue using the immunostaining as a guide, with the software calculating the ratio of isotope labelled standard to endogenous cholesterol in that chosen region. The ratio was then multiplied by the density of standard on tissue (120 ng/mm^2^) to get the average relative amount of cholesterol across a given area.
